# 4-[(*E*)-(2,3-Dichloro­benzyl­idene)amino]­phenol

**DOI:** 10.1107/S1600536811019933

**Published:** 2011-06-04

**Authors:** Li-Xia Sun, Yun-Dan Yu, Guo-Ying Wei

**Affiliations:** a College of Materials Science & Engineering, China Jiliang University, Hangzhou 310018, People’s Republic of China

## Abstract

In the title compound, C_13_H_9_Cl_2_NO, the dihedral angle between the benzene rings is 54.22 (10)°. In the crystal, mol­ecules are linked by O—H⋯N inter­molecular hydrogen bonds, forming a zigzag *C*(7) chain along the *a* axis.

## Related literature

For the biological properties of Schiff base ligands, see: Bedia *et al.* (2006 ▶). For related structures, see: Fun *et al.* (2008 ▶); Alhadi *et al.* (2008 ▶); Nie (2008 ▶). For reference bond-length values, see: Allen *et al.* (1987 ▶).
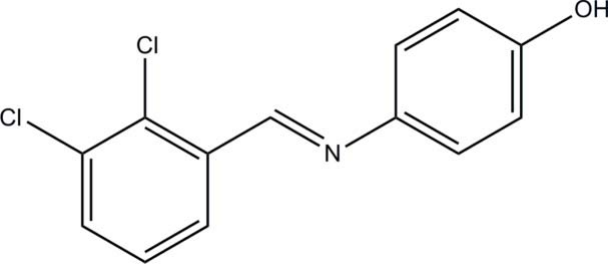


## Experimental

### 

#### Crystal data

C_13_H_9_Cl_2_NO
*M* *_r_* = 266.11
Orthorhombic, 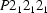

*a* = 6.049 (4) Å
*b* = 10.038 (6) Å
*c* = 19.645 (12) Å
*V* = 1192.8 (13) Å^3^
*Z* = 4
Mo *K*α radiation
μ = 0.52 mm^−1^
*T* = 296 K
0.25 × 0.23 × 0.21 mm

#### Data collection

Bruker APEXII CCD diffractometer
Absorption correction: multi-scan (*SADABS*; Bruker, 2004 ▶) *T*_min_ = 0.880, *T*_max_ = 0.898
4853 measured reflections
2184 independent reflections
1998 reflections with *I* > 2σ(*I*)
*R*_int_ = 0.037

#### Refinement

*R*[*F* ^2^ > 2σ(*F* ^2^)] = 0.030
*wR*(*F* ^2^) = 0.072
*S* = 1.17
2184 reflections
156 parameters
H-atom parameters constrained
Δρ_max_ = 0.15 e Å^−3^
Δρ_min_ = −0.14 e Å^−3^
Absolute structure: Flack (1983 ▶), 869 Friedel pairs
Flack parameter: 0.04 (6)

### 

Data collection: *APEX2* (Bruker, 2004 ▶); cell refinement: *SAINT* (Bruker, 2004 ▶); data reduction: *SAINT*; program(s) used to solve structure: *SHELXS97* (Sheldrick, 2008 ▶); program(s) used to refine structure: *SHELXL97* (Sheldrick, 2008 ▶); molecular graphics: *SHELXTL* (Sheldrick, 2008 ▶); software used to prepare material for publication: *SHELXTL*.

## Supplementary Material

Crystal structure: contains datablock(s) global, I. DOI: 10.1107/S1600536811019933/hb5895sup1.cif

Structure factors: contains datablock(s) I. DOI: 10.1107/S1600536811019933/hb5895Isup2.hkl

Supplementary material file. DOI: 10.1107/S1600536811019933/hb5895Isup3.cml

Additional supplementary materials:  crystallographic information; 3D view; checkCIF report

## Figures and Tables

**Table 1:** Hydrogen-bond geometry (Å, °)

*D*—H⋯*A*	*D*—H	H⋯*A*	*D*⋯*A*	*D*—H⋯*A*
O1—H1⋯N1^i^	0.82	1.99	2.811 (3)	174

Symmetry code: (i) 
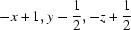
.

**Fig. 1.**
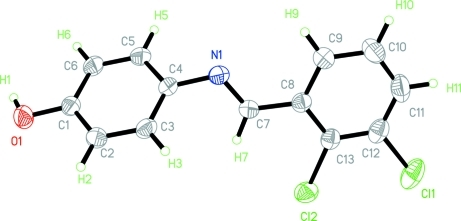
The molecular structure of the title compounds with 50% probability displacement ellipsoids for non-hydrogen atoms.

**Fig. 2.**
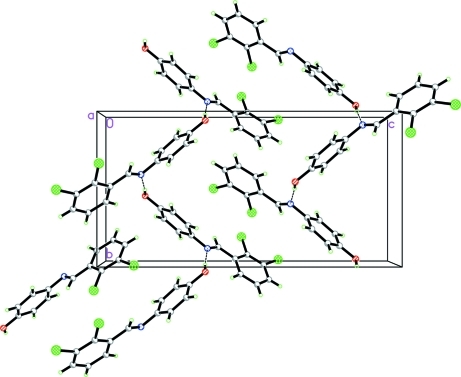
Molecular packing of the title compound, viewed along the a axis. Hydrogen bonds are shown as dashed lines.

## supplementary crystallographic information

### Comment

Schiff base ligands have received considerable attention during the last decades, mainly because of their structures or for their biological properties (Bedia *et al.*, 2006). We report here the crystal structure of the title new Schiff base compound, (I). In (I) (Fig. 1), the bond lengths and angles are normal and comparable to the values observed in similar compounds (Nie *et al.*, 2008; Fun *et al.*, 2008; Alhadi *et al.*, 2008). The dihedral angle between the two aromatic rings in the Schiff base molecule is 54.22 (10) °, indicating that two these rings are not coplanar. Intermolecular O—H···N hydrogen bonds (Table 1) link the molecules along *a* axis (Fig. 2).

### Experimental

A mixture of 2,3-dichlorobenzaldehyde (5 mmol), 4-aminophenol (5 mmol) and methanol (40 ml) was refluxed for 2 h. It was then allowed to cool and filtered. Recrystallization of the crude product from methanol yielded yellow blocks of (I).

### Refinement

H atoms were positioned geometrically and refined using the riding-model approximation, with C—H = 0.93–0.97 Å, O—H = 0.82 Å, and *U*_iso_(H) = 1.2*U*_eq_(C) or *U*_iso_(H) = 1.5*U*_eq_(O).

### Crystal data

**Table:**

C_13_H_9_Cl_2_NO	*F*(000) = 544
*M**_r_* = 266.11	*D*_x_ = 1.482 Mg m^−^^3^
Orthorhombic, *P*2_1_2_1_2_1_	Mo *K*α radiation, λ = 0.71073 Å
Hall symbol: P 2ac 2ab	Cell parameters from 2869 reflections
*a* = 6.049 (4) Å	θ = 2.3–27.2°
*b* = 10.038 (6) Å	µ = 0.52 mm^−^^1^
*c* = 19.645 (12) Å	*T* = 296 K
*V* = 1192.8 (13) Å^3^	Block, yellow
*Z* = 4	0.25 × 0.23 × 0.21 mm

### Data collection

**Table:**

Bruker APEXII CCD diffractometer	2184 independent reflections
Radiation source: fine-focus sealed tube	1998 reflections with *I* > 2σ(*I*)
graphite	*R*_int_ = 0.037
φ and ω scans	θ_max_ = 25.5°, θ_min_ = 2.3°
Absorption correction: multi-scan (*SADABS*; Bruker, 2004)	*h* = −7→7
*T*_min_ = 0.880, *T*_max_ = 0.898	*k* = −8→12
4853 measured reflections	*l* = −23→21

### Refinement

**Table:**

Refinement on *F*^2^	Hydrogen site location: inferred from neighbouring sites
Least-squares matrix: full	H-atom parameters constrained
*R*[*F*^2^ > 2σ(*F*^2^)] = 0.030	*w* = 1/[σ^2^(*F*_o_^2^) + (0.0269*P*)^2^] where *P* = (*F*_o_^2^ + 2*F*_c_^2^)/3
*wR*(*F*^2^) = 0.072	(Δ/σ)_max_ < 0.001
*S* = 1.17	Δρ_max_ = 0.15 e Å^−^^3^
2184 reflections	Δρ_min_ = −0.14 e Å^−^^3^
156 parameters	Extinction correction: *SHELXL97* (Sheldrick, 2008), Fc^*^=kFc[1+0.001xFc^2^λ^3^/sin(2θ)]^-1/4^
0 restraints	Extinction coefficient: 0.073 (4)
Primary atom site location: structure-invariant direct methods	Absolute structure: Flack (1983), 869 Friedel pairs
Secondary atom site location: difference Fourier map	Flack parameter: 0.04 (6)

### Special details

**Table:**

Geometry. All e.s.d.'s (except the e.s.d. in the dihedral angle between two l.s. planes) are estimated using the full covariance matrix. The cell e.s.d.'s are taken into account individually in the estimation of e.s.d.'s in distances, angles and torsion angles; correlations between e.s.d.'s in cell parameters are only used when they are defined by crystal symmetry. An approximate (isotropic) treatment of cell e.s.d.'s is used for estimating e.s.d.'s involving l.s. planes.
Refinement. Refinement of *F*^2^ against ALL reflections. The weighted *R*-factor *wR* and goodness of fit *S* are based on *F*^2^, conventional *R*-factors *R* are based on *F*, with *F* set to zero for negative *F*^2^. The threshold expression of *F*^2^ > σ(*F*^2^) is used only for calculating *R*-factors(gt) *etc*. and is not relevant to the choice of reflections for refinement. *R*-factors based on *F*^2^ are statistically about twice as large as those based on *F*, and *R*- factors based on ALL data will be even larger.

### Fractional atomic coordinates and isotropic or equivalent isotropic displacement parameters (Å^2^)

**Table:**

	*x*	*y*	*z*	*U*_iso_*/*U*_eq_	
C1	0.6782 (4)	0.13161 (18)	0.30064 (9)	0.0315 (5)	
C2	0.8717 (4)	0.2032 (2)	0.29247 (10)	0.0380 (5)	
H2	0.9883	0.1903	0.3226	0.046*	
C3	0.8932 (4)	0.2937 (2)	0.23991 (10)	0.0353 (5)	
H3	1.0241	0.3413	0.2347	0.042*	
C4	0.7192 (3)	0.31355 (18)	0.19496 (10)	0.0305 (5)	
C5	0.5261 (4)	0.2441 (2)	0.20450 (11)	0.0389 (5)	
H5	0.4074	0.2593	0.1754	0.047*	
C6	0.5046 (4)	0.1522 (2)	0.25642 (11)	0.0380 (5)	
H6	0.3739	0.1045	0.2615	0.046*	
C7	0.9040 (4)	0.3995 (2)	0.10174 (10)	0.0305 (5)	
H7	1.0160	0.3406	0.1138	0.037*	
C8	0.9356 (3)	0.48183 (19)	0.04113 (10)	0.0302 (5)	
C9	0.7862 (4)	0.5815 (2)	0.02412 (11)	0.0404 (5)	
H9	0.6685	0.5997	0.0531	0.049*	
C10	0.8097 (4)	0.6539 (2)	−0.03507 (13)	0.0528 (6)	
H10	0.7081	0.7203	−0.0457	0.063*	
C11	0.9837 (5)	0.6281 (2)	−0.07860 (12)	0.0502 (6)	
H11	0.9987	0.6761	−0.1188	0.060*	
C12	1.1338 (4)	0.5315 (2)	−0.06226 (10)	0.0392 (5)	
C13	1.1149 (4)	0.45927 (17)	−0.00259 (10)	0.0317 (5)	
Cl1	1.35264 (12)	0.50375 (7)	−0.11774 (3)	0.0628 (2)	
Cl2	1.31056 (9)	0.33967 (5)	0.01639 (3)	0.04412 (18)	
N1	0.7322 (3)	0.40395 (17)	0.13882 (8)	0.0321 (4)	
O1	0.6681 (3)	0.04560 (15)	0.35434 (8)	0.0447 (4)	
H1	0.5510	0.0045	0.3531	0.067*	

### Atomic displacement parameters (Å^2^)

**Table:**

	*U*^11^	*U*^22^	*U*^33^	*U*^12^	*U*^13^	*U*^23^
C1	0.0295 (12)	0.0359 (10)	0.0290 (10)	−0.0003 (9)	0.0016 (9)	0.0003 (8)
C2	0.0288 (12)	0.0525 (12)	0.0326 (11)	−0.0050 (10)	−0.0052 (9)	0.0053 (9)
C3	0.0249 (12)	0.0454 (12)	0.0355 (11)	−0.0058 (10)	0.0016 (9)	0.0011 (9)
C4	0.0271 (12)	0.0343 (10)	0.0302 (10)	0.0027 (9)	0.0046 (8)	0.0020 (8)
C5	0.0230 (12)	0.0556 (14)	0.0382 (12)	0.0002 (10)	−0.0027 (10)	0.0068 (10)
C6	0.0241 (11)	0.0480 (12)	0.0419 (12)	−0.0102 (11)	0.0010 (9)	0.0070 (10)
C7	0.0270 (11)	0.0325 (10)	0.0319 (11)	0.0014 (9)	−0.0015 (9)	0.0009 (8)
C8	0.0271 (11)	0.0311 (10)	0.0323 (10)	−0.0043 (9)	−0.0019 (8)	−0.0004 (8)
C9	0.0368 (13)	0.0421 (11)	0.0424 (12)	0.0024 (10)	0.0006 (11)	0.0075 (10)
C10	0.0516 (16)	0.0468 (13)	0.0601 (15)	0.0043 (13)	−0.0085 (13)	0.0184 (12)
C11	0.0582 (17)	0.0521 (14)	0.0403 (13)	−0.0114 (13)	−0.0060 (12)	0.0166 (11)
C12	0.0396 (14)	0.0467 (12)	0.0312 (11)	−0.0145 (11)	0.0007 (10)	−0.0038 (9)
C13	0.0323 (12)	0.0321 (10)	0.0309 (11)	−0.0072 (8)	−0.0015 (9)	−0.0021 (8)
Cl1	0.0631 (5)	0.0830 (5)	0.0424 (4)	−0.0188 (4)	0.0200 (3)	−0.0021 (3)
Cl2	0.0381 (3)	0.0505 (3)	0.0437 (3)	0.0092 (3)	0.0070 (3)	−0.0034 (2)
N1	0.0269 (10)	0.0360 (9)	0.0334 (9)	0.0021 (7)	0.0006 (8)	0.0007 (7)
O1	0.0372 (10)	0.0563 (9)	0.0407 (8)	−0.0109 (8)	−0.0033 (7)	0.0166 (7)

### Geometric parameters (Å, °)

**Table:**

C1—O1	1.365 (2)	C7—H7	0.9300
C1—C6	1.379 (3)	C8—C9	1.390 (3)
C1—C2	1.382 (3)	C8—C13	1.402 (3)
C2—C3	1.382 (3)	C9—C10	1.378 (3)
C2—H2	0.9300	C9—H9	0.9300
C3—C4	1.388 (3)	C10—C11	1.381 (4)
C3—H3	0.9300	C10—H10	0.9300
C4—C5	1.373 (3)	C11—C12	1.366 (3)
C4—N1	1.430 (2)	C11—H11	0.9300
C5—C6	1.381 (3)	C12—C13	1.383 (3)
C5—H5	0.9300	C12—Cl1	1.737 (2)
C6—H6	0.9300	C13—Cl2	1.727 (2)
C7—N1	1.270 (3)	O1—H1	0.8200
C7—C8	1.462 (3)		
			
O1—C1—C6	123.22 (19)	C9—C8—C13	118.18 (19)
O1—C1—C2	117.18 (18)	C9—C8—C7	121.20 (19)
C6—C1—C2	119.58 (18)	C13—C8—C7	120.60 (18)
C3—C2—C1	120.57 (19)	C10—C9—C8	121.0 (2)
C3—C2—H2	119.7	C10—C9—H9	119.5
C1—C2—H2	119.7	C8—C9—H9	119.5
C2—C3—C4	119.86 (19)	C9—C10—C11	120.1 (2)
C2—C3—H3	120.1	C9—C10—H10	119.9
C4—C3—H3	120.1	C11—C10—H10	119.9
C5—C4—C3	119.06 (18)	C12—C11—C10	119.6 (2)
C5—C4—N1	118.29 (18)	C12—C11—H11	120.2
C3—C4—N1	122.66 (17)	C10—C11—H11	120.2
C4—C5—C6	121.3 (2)	C11—C12—C13	121.1 (2)
C4—C5—H5	119.3	C11—C12—Cl1	118.21 (17)
C6—C5—H5	119.3	C13—C12—Cl1	120.71 (18)
C1—C6—C5	119.6 (2)	C12—C13—C8	119.9 (2)
C1—C6—H6	120.2	C12—C13—Cl2	119.40 (17)
C5—C6—H6	120.2	C8—C13—Cl2	120.68 (15)
N1—C7—C8	123.66 (19)	C7—N1—C4	117.70 (17)
N1—C7—H7	118.2	C1—O1—H1	109.5
C8—C7—H7	118.2		
			
O1—C1—C2—C3	−179.00 (19)	C9—C10—C11—C12	−0.8 (4)
C6—C1—C2—C3	−0.8 (3)	C10—C11—C12—C13	−0.2 (3)
C1—C2—C3—C4	0.2 (3)	C10—C11—C12—Cl1	−179.07 (19)
C2—C3—C4—C5	1.3 (3)	C11—C12—C13—C8	2.0 (3)
C2—C3—C4—N1	−178.85 (18)	Cl1—C12—C13—C8	−179.16 (15)
C3—C4—C5—C6	−2.1 (3)	C11—C12—C13—Cl2	−179.20 (17)
N1—C4—C5—C6	177.97 (19)	Cl1—C12—C13—Cl2	−0.4 (2)
O1—C1—C6—C5	178.0 (2)	C9—C8—C13—C12	−2.8 (3)
C2—C1—C6—C5	0.0 (3)	C7—C8—C13—C12	175.34 (18)
C4—C5—C6—C1	1.5 (3)	C9—C8—C13—Cl2	178.46 (15)
N1—C7—C8—C9	6.8 (3)	C7—C8—C13—Cl2	−3.4 (3)
N1—C7—C8—C13	−171.26 (19)	C8—C7—N1—C4	177.33 (18)
C13—C8—C9—C10	1.8 (3)	C5—C4—N1—C7	−133.5 (2)
C7—C8—C9—C10	−176.3 (2)	C3—C4—N1—C7	46.7 (3)
C8—C9—C10—C11	−0.1 (4)		

### Hydrogen-bond geometry (Å, °)

**Table:**

*D*—H···*A*	*D*—H	H···*A*	*D*···*A*	*D*—H···*A*
O1—H1···N1^i^	0.82	1.99	2.811 (3)	174

Symmetry codes: (i) −*x*+1, *y*−1/2, −*z*+1/2.
